# Controlling infectious disease through the targeted manipulation of contact network structure

**DOI:** 10.1016/j.epidem.2015.02.008

**Published:** 2015-09

**Authors:** M. Carolyn Gates, Mark E.J. Woolhouse

**Affiliations:** Epidemiology Group, Centre for Immunity, Infection and Evolution, School of Biological Sciences, University of Edinburgh, Ashworth Laboratories, Kings Buildings, West Mains Road, Edinburgh EH9 3JT, UK

**Keywords:** Network analysis, Cattle movements, Configuration wiring, Disease control

## Abstract

Individuals in human and animal populations are linked through dynamic contact networks with characteristic structural features that drive the epidemiology of directly transmissible infectious diseases. Using animal movement data from the British cattle industry as an example, this analysis explores whether disease dynamics can be altered by placing targeted restrictions on contact formation to reconfigure network topology. This was accomplished using a simple network generation algorithm that combined configuration wiring with stochastic block modelling techniques to preserve the weighted in- and out-degree of individual nodes (farms) as well as key demographic characteristics of the individual network connections (movement date, livestock market, and animal production type). We then tested a control strategy based on introducing additional constraints into the network generation algorithm to prevent farms with a high in-degree from selling cattle to farms with a high out-degree as these particular network connections are predicted to have a disproportionately strong role in spreading disease. Results from simple dynamic disease simulation models predicted significantly lower endemic disease prevalences on the trade restricted networks compared to the baseline generated networks. As expected, the relative magnitude of the predicted changes in endemic prevalence was greater for diseases with short infectious periods and low transmission probabilities. Overall, our study findings demonstrate that there is significant potential for controlling multiple infectious diseases simultaneously by manipulating networks to have more epidemiologically favourable topological configurations. Further research is needed to determine whether the economic and social benefits of controlling disease can justify the costs of restricting contact formation.

## Highlights

• Contact network structure has a strong influence on disease transmission dynamics.
• We develop a novel algorithm to reconfigure important network structural properties.
• Data from cattle movement networks in the United Kingdom is used as an example.
• Our approach preserves key demographic characteristics of between-herd contacts.
• Preventing formation of high risk contacts can substantially alter disease dynamics.

## 1 Introduction

Human and animal populations are susceptible to a wide range of infectious diseases that spread between individuals through everyday social interactions. Until recently, it was common practice to model these contact patterns using mass-action mixing approaches, which assume that individuals in a population all make the same number of contacts and mix homogeneously such that the probability of any two individuals forming a connection is always equal. However, it has now been well-established that the contact distribution is highly right skewed, meaning that a small number of individuals make a disproportionately large number of contacts, and that the contacts themselves organize into complex networks with particular structural features that determine how far and how fast disease can spread within the population (Keeling, 2005, Shirley and Rushton, 2005), For example, the highly right skewed contact distribution leads to the emergence of scale-free behaviour characterized by epidemic thresholds that converge to zero (Barabasi, 2009), higher basic reproduction numbers (*R*_0_) than expected for networks with uniform degree distributions(Woolhouse et al., 2005), and greater tolerance to disease control measures that are applied at random (Albert et al., 2000). Many biological networks also display small-world properties characterized by the local clustering of contacts with the occasional long distance jumps that are responsible for spreading disease to more distant network communities (Watts and Strogatz, 1998).

From an epidemiological perspective, it has consistently been reported that removing the small number of highly connected individuals or highly central network connections is the most cost-effective means of controlling disease at the population level (Christley et al., 2005, Kiss et al., 2006, Munro and Gregory, 2009, Pautasso et al., 2009, Rautureau et al., 2012). There are, however, several practical limitations to this approach. First, any intervention that involves physically removing high risk individuals or contacts from the network, such as the use of school closures to mitigate influenza pandemics (Cauchemez et al., 2009), can have significant economic and social costs. It can also be difficult to identify these individuals or contacts without full knowledge of the contact network structure (Kitsak et al., 2010). Second, interventions that involve effectively removing high risk individuals or contacts from the network require there to be an effective vaccination, treatment, or quarantine protocol for the disease of interest. Many infectious diseases of cattle, for example, have a subclinical carrier state for which there are few reliable ante-mortem diagnostic tests or preventive vaccinations and for which quarantine or treatment are unlikely to prevent disease introductions (Lindberg and Alenius, 1999; de la Rua-Domenech et al., 2006; Nielsen and Toft, 2008). Furthermore, these measures are often costly and highly pathogen specific, which leads to necessary trade-offs in resource allocation. As highlighted in a recent review (Carslake et al., 2011), it is important to develop control strategies that are effective against multiple infectious diseases simultaneously.

Numerous theoretical modelling studies have shown that the transmission dynamics of many infectious diseases can be altered by modifying specific structural properties of the contact network. For example, increasing network clustering almost invariably slows epidemic spread due to the rapid depletion of local susceptible contacts (Holme and Kim, 2002, Newman, 2003, Volz et al., 2011). In assortative networks where highly connected individuals form contacts with other highly connected individuals, disease tends to spread more rapidly (Newman, 2002, Kiss et al., 2008), but the probability of extinction is greater (Nishiura et al., 2011) and fewer individuals become infected over the course of the epidemic (Badham and Stocker, 2010). Other researchers have also explored the effects of homogenizing the degree distribution (May and Lloyd, 2001, Pastor-Satorras and Vespignani, 2002, Ames et al., 2011) or introducing higher order community structures (Liu and Bambi, 2005, Salathé and Jones, 2010). However, it is difficult to determine whether these observations have practical value for disease control since the network generation models often use arbitrary rules and scaling constants to generate the desired structural properties. For example, preferential attachment has been used as a mechanism to explain scale-free degree distributions (Barabasi and Albert, 1999). In this system, the probability of a new connection forming with a node is significantly greater if the node already has other network contacts. Additional rules can be introduced to increase the likelihood that any two neighbours of a node will also form connections, thereby creating networks with specified clustering coefficients (Newman, 2001, Holme and Kim, 2002, Vazquez, 2003). In biological systems, there are inherent constraints to contact formation such as geographical distance, social preferences, and seasonality that limit the potential variation in network topology (Mahmood et al., 2010).

An alternative approach is to generate contact networks from first principles based on a sound understanding of the biological, social, and financial factors driving contact formation (Mahmood et al., 2010). Recent work has demonstrated that it is possible to replicate human mobility patterns from information on job opportunities and the assumption that individuals would seek to maximize income while minimizing commuting distance (Simini et al., 2012). In the cattle industry, high transportation costs also result in most contacts occurring over short distances. Lindström and colleagues used this principle to construct theoretical cattle networks where the probability of contact formation was modelled as a function distance between herds and the demographic characteristics of the farm (Lindström et al., 2009, Lindström et al., 2011, Lindström et al., 2013). Stochastic block modelling, where farms are assigned into groups and contacts generated based on the probability that any two farms within a group or between groups will form a contact (Karrer and Newman, 2011), was used successfully to generate contact networks with spatial clustering to describe transmission pathways for the 2007 equine influenza outbreak in Australia (Firestone et al., 2011). However, both approaches ignored the timing of movements and the latter study ignored the production characteristics of the farm, which are important constraints to contact formation in livestock populations (Bajardi et al., 2011, Holme and Saramäki, 2012).

In this analysis, we first present a novel framework for generating contact networks from first principles that uses configuration wiring (Serrano and Boguna, 2005) to preserve the number of contacts made by individuals and stochastic block modelling (Karrer and Newman, 2011) to preserve important demographic features of the network connections. Movement data from the British cattle industry is used for illustrative purposes. Cattle farms are highly constrained in the number of animals that must be bought or sold each year to meet production needs and contacts can only form between farms that are trading the same production type of cattle through the same livestock markets at the same time of year. We then show how the basic network generation algorithm can be modified to test control strategies that minimize the probability of forming contacts with a disproportionately strong risk of spreading disease through the network. Our results are used to the highlight the potential for controlling infectious disease in human and animal populations by intentionally engineering contact networks to have more epidemiologically favourable structural features.

## 2 Materials and methods

### 2.1 Cattle movement data

Records of the births, deaths, and movements of individual cattle in Great Britain have been stored in the electronic Cattle Tracing System (CTS) database operated by the British Cattle Movement Service (BCMS) since 1998 (Mitchell et al., 2005). This database was created under European Union Council Regulation (EC) No 820/97 as part of larger efforts to restore consumer confidence in the safety of livestock products following the bovine spongiform encephalopathy (BSE) crisis in 1996 and has provided researchers with an unprecedented opportunity to generate detailed network representations of industry contact patterns (Kao et al., 2006, Green et al., 2008, Keeling et al., 2010, Tinsley et al., 2012). Each movement record contains basic information on the animal identification number, the departure location type and identification number, the destination location type and identification number, and the movement date. This may be linked with demographic information for each animal (including the sex, breed, birth date, death date, and any previously or subsequently recorded calvings) to infer its production purpose at the time of movement (Gates, 2014).

An extract of the CTS database containing all known records through April 2010 was provided by the Department for Environment, Food, and Rural Affairs (DEFRA). This analysis focused on the subset of all individual beef and dairy cattle movements between locations classified as agricultural holdings, landless keepers (farmers that raise cattle on rented land), and livestock markets during the 2006 calendar year. The reason for selecting this year was so that sufficient pre- and post-movement data was available to classify animals into production groups at the time of movement. As the primary focus of this analysis was on the spread of disease through livestock trade, we excluded movements to locations classified as abattoirs, showgrounds, and artificial insemination centres. Movements to abattoirs represent a dead end for disease transmission, while small number of movements to showgrounds and artificial insemination centres (less than 0.5% of all individual cattle movements) represent a temporary relocation of cattle and are also believed to have a negligible role in disease transmission (Volkova et al., 2010a). For movements that occurred through a livestock market, the movement record onto the market was paired with the corresponding movement record off the market to preserve the identity of the source and destination herds.

The final data set contained records of 2,609,576 individual animal movements. Animals were classified into one of the following three production groups based on their demographic characteristics at the time of movement: beef breeding female, dairy breeding female, and store cattle (including male store cattle, female store cattle, and an unknown number of breeding bulls). The beef and dairy breeding female groups included animals with at least one recorded calving in the CTS database and animals that survived beyond 30 months of age. It was assumed that animals intended for human consumption in Great Britain would be slaughtered by 30 months of age to comply with regulations to control BSE (DEFRA, 2010). The CTS movement data was processed in the Python programming environment.

### 2.2 Network terminology

The terminology used to describe contact networks was originally derived from mathematical graph theory. In this system, a network is defined as a collection of units or *nodes* that are connected through a specified relationship or *edge.* When the relationships are unidirectional, such as the movements of cattle from a source herd to a destination herd, the network is said to be *directed*. When the relationships are bidirectional, such as the nose-to-nose contact of cattle through fencelines, the network is said to be *undirected*. Node *degree* measures how many direct contacts or edges a farm has with others in the network. Edges may be *weighted* according to the frequency of occurrence or the number of the animals moved. In directed networks, degree can be further partitioned into *in-degree* and *out-degree* representing the number of potential sources and sinks for disease transmission, respectively. The degree of separation between any given pair of nodes in the network is measured by the *path length*. Collectively, the broad patterns in how nodes and edges are arranged in the network are referred to as *network topology*. Comprehensive reviews of network analysis and its applications to veterinary medicine are provided elsewhere (Dubé et al., 2009, Martínez-López et al., 2009).

**Fig. 1:**
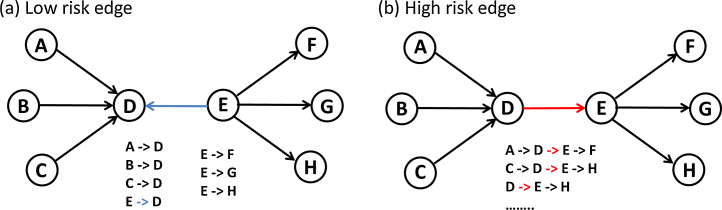
Schematic representation of network edges with disproportionately (a) low and (b) high risk of spreading disease through the network.

The relative importance of individual movements to network transmission dynamics depends in part on the connectivity of the source and destination herds. In general, a directed edge that connects a farm with a high in-degree (associated with an increased risk of acquiring disease) to a farm with a high out-degree (associated with an increased risk of spreading disease) is expected to play a greater role in propagating disease through the network (Fig. 1). The presence of these high-risk edges can be quantified through two network topology features called *degree assortativity* and *edge betweenness centrality*. Degree assortativity measures the likelihood that nodes will preferentially from connections with other nodes that have similar degree distributions (Newman, 2002). In directed networks, degree assortativity can be further partitioned into four measures (out-in disassortative, out-out disassortative, in-out assortative, and in-in assortative) based on the in- and out-degree distribution of the source and destination nodes (Foster et al., 2010). Edge betweenness centrality measures the total number of times an edge falls on the shortest path between any two farms in the network. Through dynamic simulation models, it has been consistently shown that removing a small number of edges with the highest betweenness centrality causes a disproportionately strong reduction in the transmission and prevalence of infectious livestock diseases (Kiss et al., 2006, Green et al., 2009, Rautureau et al., 2010). In this analysis, we focus on reconfiguring network topology to minimize the number of high risk connections between farms with a high in-degree and farms with a high out degree, which is expected to create more disassortative networks with lower edge betweenness centrality scores.

### 2.3 Network generation model

**Fig. 2:**
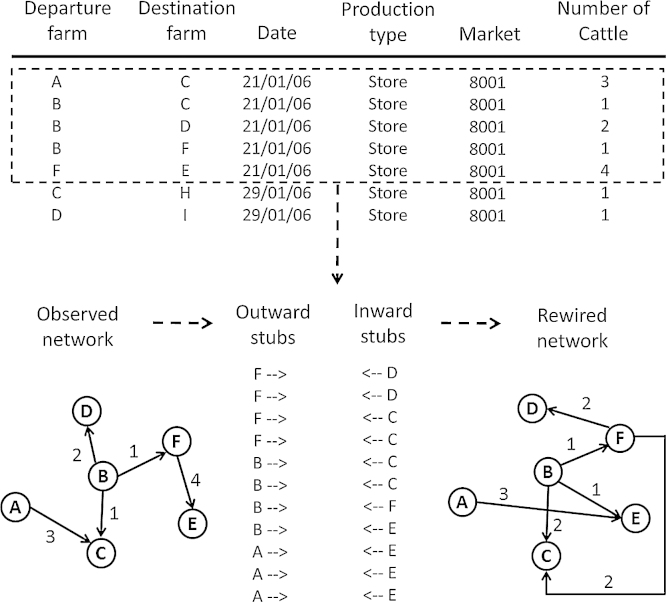
Schematic representation of the basic network generation algorithm.

The network generation model was based on the assumption that the total number of cattle purchased and sold by farms on any given day was fixed. To preserve these numbers, we used a modified version of the configuration wiring algorithm (Serrano and Boguna, 2005). Our method for introducing restrictions in the model so that edges could only be formed between farms that traded cattle of the same production type through the same market on the same day was similar to the stochastic block model described by Karrer and Newman (2011). In this system, individuals are assigned into one of *K* groups based on specified characteristics. The probability of individuals from any two groups forming a contact can be modified to produce networks with a wide variety of different structures. The objective was not to produce an exact replicate of the observed cattle movement network as this requires in-depth knowledge of the factors driving cattle trade, but rather to provide a benchmark against which to compare the effects of rewiring the movement network under different constraints. In particular, we note that our approach does not capture how animals are aggregated and sold as batches at market and that our approach is also not designed to account for connections between farms that are repeated multiple times during the year, which increases the mean degree of the network and, by extension, alters the network transmission dynamics (Ames et al., 2011). The following describes the three main steps involved in the network generation algorithm:

1. Each farm in the population was assigned a fixed number of outward “stubs” and inward “stubs” corresponding to the observed number of cattle sold and purchased on a given day. Each stub represented an individual animal and was created as a virtual object with the basic attributes of farm identification number, market location, date, and animal production type. The system was assumed to be closed such that the total number of outward stubs in the system was always equal to the total number of inward stubs. At the beginning of each network wiring simulation, the lists of outward stubs and inward stubs were first ordered by day, market, animal production type to generate the *K* groups and then randomly by farm identification number to introduce stochastic variation with each rewiring attempt.
2. Working down the lists of outward and inward stubs, connections were formed only if (i) the inward stub occurred through the same market on the same date and had the same animal production type as the outward stub and (ii) the departure farm was different from the destination farm so that no loop edges were formed. Once a connection was made, the choice of contacts for the next outward stub was inherently limited to remaining inward stubs. The process continued until all outward stubs were matched with an inward stub. With both constraints, there were approximately 1000 stubs (less than 0.04% of the total number of stubs) in each network rewiring replicate where a suitable match could not be found. We chose simply to discard these stubs since it had minimal impact on the resulting network structure. A schematic representation of the network generation algorithm is shown in Fig. 2.
3. The resulting set of match stubs was aggregated into batch movements such that all animals moved between Farm A and Farm B on a given date were considered a batch. The output from each rewiring simulation was therefore an edge set containing the departure farm identification number, destination farm identification number, movement date, and batch size.

### 2.4 Alternate rewiring algorithm

The basic network generation algorithm was modified to create networks that minimized the formation of high risk edges between source farms with a high in-degree and destination farms with a high out-degree. First, the total number of inward contacts (in-degree) and outward contacts (out-degree) was calculated for each farm during the 2006 calendar year. Then, during step 1 of the network generation algorithm, the list of start stubs was ordered by day, market, animal production type, farm in-degree descending, and farm identification number while the list of end stubs was ordered by day, market, animal production type, farm out-degree ascending, and farm identification number. The purpose for ordering the lists in this manner was to first preserve the demographic characteristics of the edge (movement date, market, and production type) and then to have farms with a high in-degree preferentially form connections to farms with a low out-degree to reduce the risk of disease transmission through the rewired edge. For farms with the same degree, the order within the list of stubs was randomized at the beginning of each rewiring simulation to introduce stochastic variation.

### 2.5 Disease simulation model

To explore the effects of rewiring on disease transmission dynamics, we used a simple susceptible-infectious-susceptible (SIS) network simulation model. Simulation modelling accounts for all the subtle changes in network structure beyond degree assortativity and edge betweenness centrality that may be contributing to the observed changes in disease transmission dynamics. At the beginning of each simulation, disease was seeded on 10% of farms at random on 01 January 2006. Each infected farm was assigned an infectious period drawn at random from an exponential distribution with a half-life of *h* days. The infection status of farms and the movements between farms were then updated in time steps of one day. If an infected farm moved cattle to a susceptible farm, there was a fixed probability, *p*, that an individual animal was infectious and the overall risk of disease transmission was the weighted probability that at least one animal in the batch was infectious. Farms that reached the end of their infectious period reverted back to a susceptible state. To ensure adequate time for the system to reach steady state equilibrium, the simulation was allowed to run for a total of 25 years by recycling the single year of movement data from the rewired networks. For simulations where the disease persisted, endemic prevalence was calculated as the average percentage of farms infected on any given day over the last year of the simulation.

To determine whether the effects of network rewiring varied based on the pathogen characteristics, a range of values for *h* and *p* were explored. At the beginning of each simulation, the value for *h* was selected at random from a uniform distribution ranging from 90 days to 1095 days and the value for *p* was selected at random from a uniform distribution ranging from 0.001 to 0.10. These parameter ranges were chosen based on how the simulated diseases behaved on the networks. Pathogens with farm infectious periods below 90 days were generally unable to persist. When the farm infectious period was increased above 1095 days or the transmission probability was increased above 0.10, the network saturated and there was little appreciable change in the endemic prevalence. A total of 10,000 simulations each were run for the baseline network generation model and the alternate network rewiring model with a new rewiring performed at the beginning of each simulation replicate to introduce additional stochastic variation. This number of simulations was adequate to capture the stochastic variation in simulation results. We then ran 10,000 simulations on the observed movement network from 2006 using the same disease parameters to evaluate how well the baseline network generation model captured network transmission dynamics. The network generation models and simulation models were both implemented in the C programming language and rigorously tested to ensure there were no coding errors.

We also performed simple disease simulations to confirm the importance of edge betweenness centrality and degree assortativity to network transmission dynamics as well as to provide a baseline for comparing our network rewiring approach to traditional control approaches focusing on ranked edge removal. Each network edge (defined here as a batch movement of cattle from farm *a* to farm *b* on a given day) in the observed network from 2006 was first assigned a betweenness centrality score calculated using the *edge.betweenness* function in the igraph network analysis library for the C programming language (Csardi and Nepusz, 2006) and a degree assortativity score calculated as the in-degree of the source farm multiplied by the out-degree of the destination farm. At the start of each simulation, edges were sorted (a) at random, (b) in descending order based on the betweenness centrality score, or (c) in descending order based on the degree assortativity score depending on the removal scenario being tested. For edges with tied betweenness centrality scores or degree assortativity scores, the order was randomized in each replicate. The proportion of edges to be removed from the network was then selected at random from a uniform distribution ranging from 0 and 0.5 and the edges were subsequently removed from the sorted lists. Using the same disease simulation model described above, the resulting endemic prevalence was estimated for a disease in the mid-range of our parameter sets with an average farm infectious period of 500 days and a transmission probability of 0.05. A total of 1000 replicates were performed for each scenario.

### 2.6 Descriptive analyses

Descriptive statistics on the frequency and seasonality of the observed cattle movements during the 2006 calendar year were provided. The median edge betweenness centrality scores and the network degree assortativity were calculated using the igraph network analysis library for the C programming language (Csardi and Nepusz, 2006) for 100 replicates each of the baseline network generation model and the alternate network rewiring model to highlight some of the structural changes associated with network rewiring. This number of replicates was chosen for computational reasons, but was adequate to capture the variation between network generation algorithms. The results from the disease simulation models were displayed as a heat maps showing the ratio of the endemic prevalence in the alternate network rewiring model compared to the endemic prevalence in the observed network (to explore potential artefacts introduced by the network rewiring process) and compared to the endemic prevalence in the baseline network generation model (to isolate the effects of network topological change) for the different parameter combinations of *h* and *p.* All descriptive analyses were performed using the R statistical software (R-Development-Core-Team, 2010).

## 3 Results

**Fig. 3:**
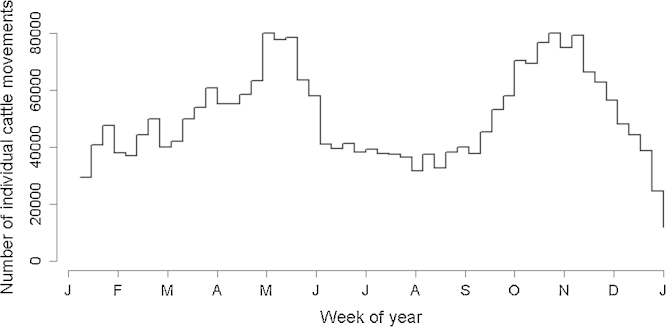
Distribution of individual cattle movements by week in the 2006 calendar year.

During the 2006 calendar year, the observed movement network contained 2,609,576 individual beef and dairy cattle movements through 691,274 network edges. There were a total of 74,987 farms and 143 locations classified as livestock markets. Although the volume of individual cattle movements through livestock markets was approximately the same as the volume of movements directly between cattle farms (53–47%), movements through markets accounted for a greater percentage of network edges (77–23%). The majority of individual movements were made by cattle classified as store animals (76%) followed by beef breeding females (13%) and dairy breeding females (11%). As shown in Fig. 3, there was a strong seasonality to cattle movements with peaks observed in late spring and early autumn.

**Fig. 4:**
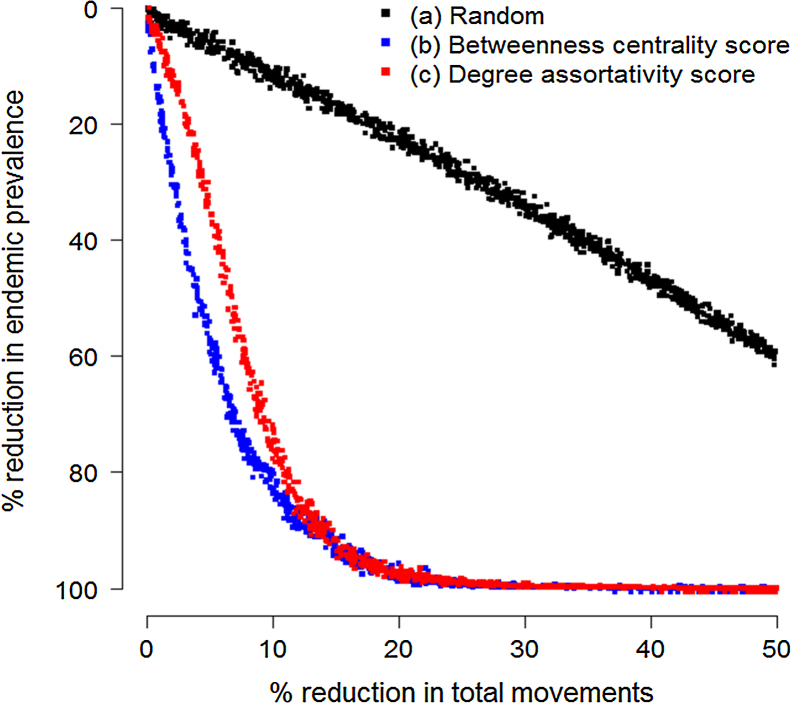
Estimated reduction in endemic prevalence following the removal of movements (a) at random, (b) ranked by betweenness centrality score, or (c) ranked by degree assortativity score.

Movements with high betweenness centrality scores and high degree assortativity scores had a disproportionately strong influence on disease transmission dynamics. For a disease with an average infectious period of 500 days and transmission probability of 0.05, removing movements ranked in the top 10% for betweenness centrality and degree assortativity reduced the endemic prevalence by over 80% (Fig. 4). In contrast, removing 10% of movements at random reduced the endemic prevalence by only 14% on average. There was a moderate and significant correlation between the betweenness centrality scores and degree assortativity scores assigned to the network edges (*r* = 0.54, *p* < 0.001).

**Fig. 5:**
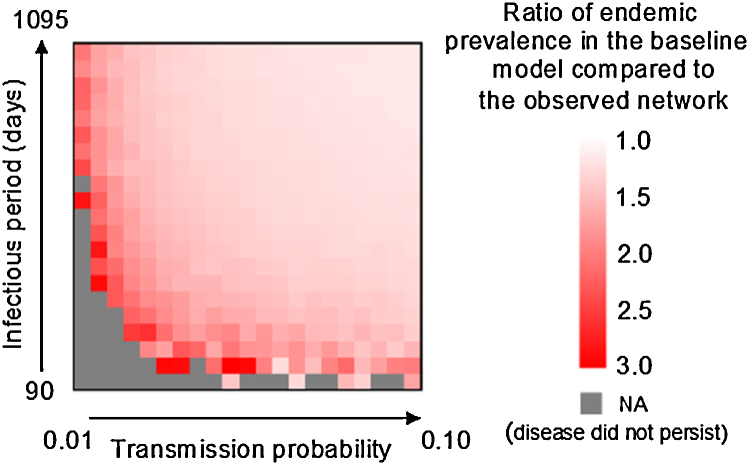
Comparison of endemic prevalences between the observed movement network and baseline network generation model. Grey squares indicate parameter combinations for which at least one simulation replicate failed to persist.

The baseline network generation model produced networks with a higher mean degree than the empirically observed contact network (11.2 versus 9.2). As a result, the predicted endemic prevalences from disease simulations on the baseline network generation model were approximately 1.2–3 times higher (median: 1.37) than for the observed network (Fig. 5). The differences were more pronounced for disease with shorter farm infectious periods and lower transmission probabilities. There was no appreciable difference in the mean degree between the baseline network generation model and the alternate rewiring model (11.2 versus 11.3).

**Fig. 6:**
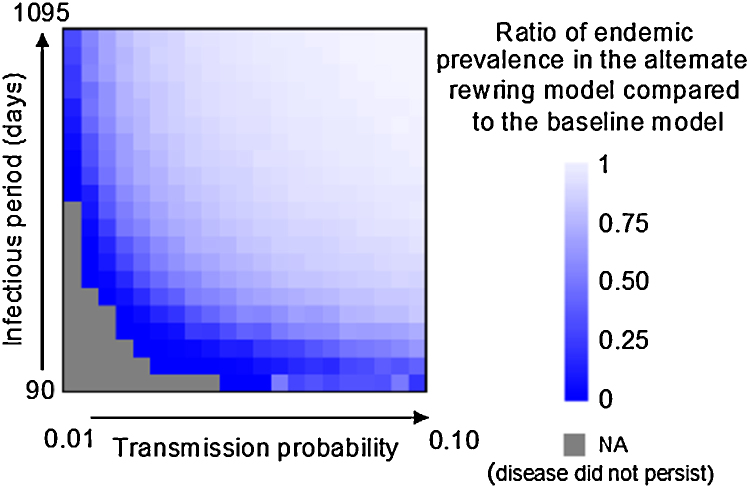
Impact of network rewiring on the predicted endemic prevalence of disease at equilibrium. Grey squares indicate parameter combinations for which at least one simulation replicate failed to persist.

As highlighted in Fig. 6, restricting contact formation between farms with a high in-degree and farms with a high out-degree in the alternate rewiring model had significant effects on the predicted endemic prevalence of the simulated cattle pathogens. The percentage reduction in endemic prevalence ranged from 4.2 to 98.3% with the strongest effects seen for pathogens with short farm infectious periods and low transmission probabilities, i.e. low *R*_0_. The median edge betweenness centrality decreased from 4741 in the baseline network generation model to 3659 in the alternate rewiring model, while the degree assortativity decreased from an average of −0.061in the baseline network generation model to −0.086 in the alternate rewiring model.

## 4 Discussion

Our study findings demonstrate that the transmission dynamics of multiple infectious cattle diseases can be altered simultaneously through movement restrictions that prevent the formation of contacts between source farms with a high in-degree and destination farms with a high out-degree. In practice, this could be implemented by assigning farms into categories based on their historically observed in-degree and out-degree distributions and then restricting trade between categories that are expected to generate high risk network edges. This strategy appears to be most effective for controlling diseases with short farm infectious periods and/or low transmission probabilities, which is not surprising given that diseases with a low *R*_0_ are expected to have a greater probability of stochastic extinction. Other researchers have similarly shown that the structural and temporal features of cattle movement networks matter less for diseases that spread over long time periods (Kao et al., 2007) or have an increased probability of spreading through batch movements (Vernon and Keeling, 2009). While we recognize that the magnitude of the effects must be interpreted with some caution given the simplifying assumptions made in both the network generation algorithm and the disease simulations, our preliminary results justify the development of more sophisticated models to weigh the financial benefits of controlling disease against the costs of trade restrictions.

For a disease with an average farm infectious period of 500 days and a transmission probability of 0.05, removing approximately 2% of individual cattle movements ranked by edge betweenness centrality or degree assortativity resulted in the same magnitude reduction in endemic prevalence as our alternate network generation model. Although it is possible that our model may perform differently for diseases with a shorter farm infectious period or lower transmission probability, it can be argued that control strategies should simply focus on edge removal rather than network reconfiguration given the smaller number of farms that would be potentially affected by trade restrictions. There are, however, practical limitations to the edge removal approach. First, edge betweenness centrality can only be calculated retrospectively by evaluating all the shortest paths between pairs of farms in the movement network. This is computationally intensive for large networks and, since most network contacts occur once without repetition (Vernon, 2011), it can be difficult to predict in advance which specific movements are most likely to have a high betweenness centrality. Second, it is difficult to reduce the total number of individual cattle traded by farms due to underlying financial and resource constraints, although recent work has shown that improving herd reproductive management may lead to reductions in replacement breeding cattle trade (Gates and Woolhouse, 2014). Third, it is difficult to effectively remove edges from the network through the use of disease specific biosecurity measures, such as testing or vaccination, which are often not available or reliable for many of the diseases spreading through cattle movement networks. Even though imposing contact restrictions can be more disruptive than targeting single nodes or edges, this control strategy has the potential to reduce the prevalence of all directly transmissible diseases spreading through the network, which may greatly offset the costs.

Although our model for generating contact networks was relatively simple, it offers advantages over the traditional ‘top down’ algorithms that rely on arbitrary rules and scaling constants to replicate features from the observed contact network (Hakansson et al., 2010). Using the configuration wiring approach, we were able to preserve the exact number of cattle purchased and sold by individual farms as well as the movement date and marketing channel. This is important for maintaining business continuity on livestock operations. To our knowledge, there are only two other published network generation models for livestock movements have reproduced the degree distribution without accounting for the number of animals traded through the contact or the movement date (Lindström et al., 2011, Lindström et al., 2013). Our baseline network generation model did however produce a greater number of network edges compared to the observed movement network from 2006. This is partly attributable to some edges between farms in the empirically observed network being repeated multiple times within the same calendar year (Vernon, 2011). These were most likely movements to and from seasonal grazing pastures, movements between uniquely identified land parcels owned by the same livestock business, or movements through premises acting as livestock dealers, although it was not possible to confirm this with the available CTS data. There are also complex factors determining how cattle shipments are divided into batches and sold at auction markets (Robinson and Christley, 2007), which were not explicitly captured in the rewiring process. Further research into livestock marketing practices will be important for improving future versions of the network generation model.

The general approach of generating contact networks from first principles is rapidly gaining traction in the social network analysis field because of the need for accurate representations of human contact patterns to feed into disease simulation models (Mahmood et al., 2010, Simini et al., 2012). This is not unlike the situation faced by many countries without centralized livestock movement recording systems. For example, researchers in the United States recently generated synthetic cattle movement networks from data collected on interstate certificates of veterinary inspection to evaluate the spread of high-impact foreign animal disease outbreaks (Buhnerkempe et al., 2014). Our network generation model can potentially be adapted to use estimates of cattle movement numbers from farmer surveys and provide better representations of livestock contact structures than are currently available. There is also a much wider range of control strategies that can be tested by modifying the stochastic block modelling component of the rewiring algorithm. For example, it may be possible to generate networks that are more spatially clustered by weighting the probability of contact formation by distance between farms or by restricting trade between farms in different network communities (Salathé and Jones, 2010). These strategies may be particularly important for controlling diseases like bovine tuberculosis (Green et al., 2008), bovine viral diarrhoea virus (Ersbøll et al., 2010), or foot-and-mouth disease (Gibbens et al., 2001) that spread through local transmission mechanisms in addition to cattle movements. Other potential strategies may include restricting trade between nodes with specific demographic characteristics to modify network structural properties such as fragmentation, assortativity, and clustering that are also known to influence transmission dynamics. It should be noted, however, that it is virtually impossible to change any single network property in isolation and the resulting changes in endemic prevalence may be apparent in multiple measures. It is also worth noting that the estimates of effect from our network reconfiguration approach may be conservative due to the strict rules that cattle farm could only trade with farms that purchased or sold cattle through the same marketing channel on the same date. Relaxing these constraints may allow networks with more favourable epidemiological features to be created and lead to greater reductions in disease prevalence.

For these types of models to be useful in guiding future policy decisions, there are several additional layers of complexity that must also be considered. First, although contact formation was restricted by basic animal production type, farmers also choose to purchase cattle based on their specific breed, age, physical condition, and reproductive status. This inherently reduces the number of potential trading partners in the network, which may in turn reduce the extent that network structure can be changed through targeted movement restrictions. Second, by recycling the single year of rewired movement data in the disease simulation model, it was assumed that the number of contacts made by individual farms and the contact patterns remained fixed over time. However, both can change quite substantially from year to year, which may provide new transmission pathways that allow disease to spread more efficiently between individuals (Volkova et al., 2010a, Vernon, 2011). New methods are currently being developed to predict a cattle farm's future epidemic risk based on past movement behaviour (Valdano et al., 2014). Third, any interventions that impose financial and logistical constraints to cattle trade may result in farmer behavioural adaptations that can have positive (Gates et al., 2013), negative (Robinson et al., 2007), or mixed (Vernon and Keeling, 2012) effects on disease transmission dynamics, which may reduce the efficacy of targeted movement restrictions. Similar challenges have been reported with modelling human behavioural response to influenza control measures (Funk et al., 2010, Cornforth et al., 2011, Poletti et al., 2011). Finally, movement restrictions can have a significant impact on farm profitability, especially if farmers must invest more time or travel further distances to trade their cattle. There is a strong need to integrate economic models with the traditional epidemiological models to provide estimates of the relative costs and benefits of different control strategies.

Our analysis used British cattle movement networks as a case example both because detailed information on the industry contact patterns was readily available through the CTS database and because contact formation in these kinds of trade networks can be manipulated through national animal health legislation. In the United Kingdom, movement standstill legislation is currently used to temporally fragment the network by preventing farms from moving animals off the premises within a specified number of days from the last animal movement onto the premises (Department for Environment, 2003DEFRA, 2003). Legislation requiring the pre- and post-movement testing of cattle from regions with a high incidence of bovine tuberculosis has also had the unintended effect of reducing the number of connections from high to low risk regions (Christley et al., 2011, Gates et al., 2013). It is likely that similar epidemiological benefits from manipulating network structure can be realized in other biological trade networks such as plant networks (Jeger et al., 2007), sheep networks (Volkova et al., 2010b), swine networks (Büttner et al., 2013), and poultry networks (Dent et al., 2008). Limiting disease transmission in the swine and poultry networks is particularly important from a public health perspective because of the potential for influenza outbreaks to spill over into human populations (Peiris et al., 2001, Koopmans et al., 2004). The opportunities for manipulating the structure of human social networks to control disease are inherently more limited. However, there are potential applications of our methodological framework for redesigning healthcare networks to prevent the spread of nosocomial infections between wards within a given hospital and between referral hospitals in the broader healthcare network (Ueno and Masuda, 2008).

## 5 Conclusions

There is significant potential to reduce the prevalence of multiple infectious diseases simultaneously by imposing restrictions on contact formation to alter key structural properties of biological networks. Future research efforts should be geared towards developing more sophisticated epidemiological models to determine whether the economic and social benefits of controlling infectious disease outweigh the costs of constraining social interactions.
